# Crosstalk between Opioid and Anti-Opioid Systems: An Overview and Its Possible Therapeutic Significance

**DOI:** 10.3390/biom10101376

**Published:** 2020-09-28

**Authors:** Ewa Gibula-Tarlowska, Jolanta H. Kotlinska

**Affiliations:** Department of Pharmacology and Pharmacodynamics, Medical University, 20-059 Lublin, Poland; jolanta.kotlinnska@umlub.pl

**Keywords:** anti-opioids, NPFF, cholecystokinin, nociceptin, MIF-1, kissorphin

## Abstract

Opioid peptides and receptors are broadly expressed throughout peripheral and central nervous systems and have been the subject of intense long-term investigations. Such studies indicate that some endogenous neuropeptides, called anti-opioids, participate in a homeostatic system that tends to reduce the effects of endogenous and exogenous opioids. Anti-opioid properties have been attributed to various peptides, including melanocyte inhibiting factor (MIF)-related peptides, cholecystokinin (CCK), nociceptin/orphanin FQ (N/OFQ), and neuropeptide FF (NPFF). These peptides counteract some of the acute effects of opioids, and therefore, they are involved in the development of opioid tolerance and addiction. In this work, the anti-opioid profile of endogenous peptides was described, mainly taking into account their inhibitory influence on opioid-induced effects. However, the anti-opioid peptides demonstrated complex properties and could show opioid-like as well as anti-opioid effects. The aim of this review is to detail the phenomenon of crosstalk taking place between opioid and anti-opioid systems at the in vivo pharmacological level and to propose a cellular and molecular basis for these interactions. A better knowledge of these mechanisms has potential therapeutic interest for the control of opioid functions, notably for alleviating pain and/or for the treatment of opioid abuse.

## 1. Introduction

The discovery of the endogenous opioid system in the 1970s initiated a new understanding of the mechanisms involved in the activity of both extracted and synthetic opium compounds. In 1973, the specific opioid binding sites in the brain were established, which became referred to as μ (μ1, μ2, μ3), δ (δ1, δ2, δ3), and κ (κ1, κ2, κ3) opioid receptors (also known, respectively, as MOR, DOR and KOR) [1,2]. The opioid receptors are classified as membrane receptors with a seven-transmembrane topology and belong to the large G protein-coupled receptor superfamily [3]. Of note, the nociceptin/orphanin FQ (N/OFQ) receptor, discovered in 1995, is currently considered to be a non-opioid branch of the opioid receptor family [1] (Table 1).

Binding sites for the main three opioid receptors overlap in most structures, but some exhibit higher expression of one receptor over the others. Opioid receptors are located in areas involved in the following: (1) pain transmission, such as the thalamus, rostroventral medulla, periaqueductal grey area, pons, or in the spinal cord of the dorsal horn; (2) the rewarding system, such as the nucleus accumbens (NAc), ventral tegmental area (VTA), or the prefrontal cortex; (3) other brain areas, such as the hypothalamus, amygdala, ventral pallidum, globus pallidus, nucleus raphe, hippocampus, and olfactory bulb [4]. However, they are also presented in peripheral tissues, for example, in the gastrointestinal and respiratory tract [4,5]. Through the characteristic distribution of opioid receptors, the opioid system plays a central role in nociception and analgesia [6,7], and it is implicated in the motivational and rewarding effects of natural rewards and the drugs of abuse. Moreover, it regulates numerous physiological actions, including responses to stress, respiration, gastrointestinal transit, as well as endocrine and immune functions [1].

In reference to the discovery of opioid receptors, several endogenous ligands forming the opioid peptide family were characterized [8]. Enkephalins, dynorphins, endorphins, and endomorphins are produced by the proteolytic cleavage of large protein precursors known as preproenkephalin (PENK), preprodynorphin (PDYN), and proopiomelanocortin (POMC), respectively. However, the exact pre-propeptide precursors of endomorphins have not yet been identified. All opioid peptides share a common NH2-terminal Tyr-Gly-Gly-Phe signature sequence, which interacts with opioid receptors [9]. Enkephalins ([Met]-enkephalin and [Leu]-enkephalin) produce the effect mainly by DOR activation, but they also have an affinity for MOR. Dynorphins (Dynorphin A and Dynorphin B) exert their effects primarily through the KOR and have less affinity for the MOR and DOR. In contrast, endorphins (α-endorphin, β-endorphin, and γ-endorphin) and endomorphins (Endomorphin-1 and Endomorphin-2) demonstrate the highest affinity and selectivity for the MOR [10] (Table 2).

There is also evidence for the existence of an endogenous anti-opioid system that balances the actions of endogenous opioids and acts as part of an endogenous homeostatic system. The first demonstration of peptide with an anti-opioid activity in 1979 and prediction of the existence of other endogenous anti-opioid peptides in the central nervous system have prompted extensive research. Presently, this group includes melanocyte inhibiting factor (MIF)-related peptides, cholecystokinin (CCK), nociceptin/orphanin (N/OFQ), neuropeptide FF (NPFF), and several others.

## 2. Opioid System and Reward

The endogenous opioid system is not only the main target for the reinforcing and rewarding effects of opioids. It is also the target of non-opioid drugs such as ethanol, cocaine, and nicotine. Research has established that by attaching to the MOR, drugs of abuse indirectly stimulate the dopaminergic neurons by inhibiting GABAergic neurons that normally maintain the mesolimbic dopamine system under an inhibitory control [11]. DOR and their endogenous ligands (to a lesser extent) are implicated in indirect positive reinforcement and the regulation of drug consumption by improving emotional states or facilitating drug-context association [12]. Likewise, the important role of KOR/dynorphin in the dysphoric effects of drugs of abuse has been thoroughly reviewed [13,14]. The obtained data indicate that PDYN-derived peptides interact with KOR mainly to limit drug reward and to conciliate the dysphoric aspects of some drugs (e.g., cannabinoids, nicotine). Moreover, under stressful conditions, KOR/dynorphin activity increases sensitivity to cocaine reward and regulates ethanol intake. Therefore, it can be concluded that the KOR activation opposes MOR signaling in the control of hedonic homeostasis and mediates the aversive effects of abused drugs observed as dysphoria, hallucinations, or malaise. Conversely, the inhibition of MOR and DOR suppresses the positive reinforcing properties of natural rewards, opiates, or non-opioid drugs, whereas the activation of KOR facilitates these effects [15,16].

## 3. Opioid System and Drugs of Abuse

**Figure 1:**
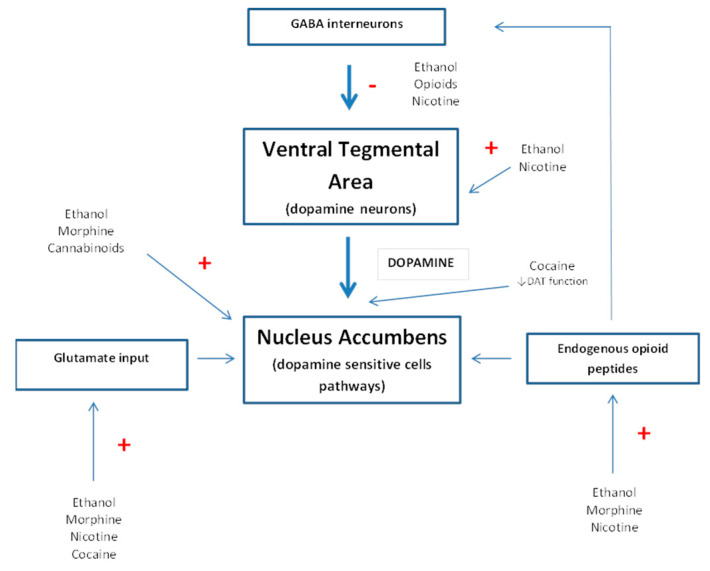
Proposed model for the pharmacological action of drugs of abuse in the ventral tegmental area–nucleus accumbens pathway. DAT: dopamine transporter.

The endogenous opioid system is central to addiction. Research on this system has substantially contributed to our understanding of the brain substrates and molecular mechanisms of drugs of abuse. Early studies focused on the role of opioid receptors as a mediator of the pharmacological effects of morphine and other opiates [17]. Subsequently, it was noticed that opioid receptors are also involved in the multiple effects of non-opioid drugs [18] (Figure 1).

Ethanol, in contrast, does not have a specific receptor, but it interacts with various receptors, neurotransmitters, and neuromodulators. However, the participation of opioid receptors in ethanol reinforcement has generated considerable interest [19,20]. As a result of such research, it was recognized that (1) opioid receptors are an important target of ethanol action in the central nervous system, (2) indirect effects of ethanol on opioid receptors contribute to its behavioral effects in animals and humans, (3) ethanol increases β-endorphin and enkephalin levels in the NAc, (4) ethanol administration causes alterations in the number and activity of opioid receptors, and (5) ethanol intake depends on the activity of the endogenous opioid system [21,22,23,24,25,26,27]. However, a growing body of evidence suggests that for the most part, MORs [28] and the activation of β-endorphinergic neurons of the arcuate nucleus are involved in the rewarding (addictive) effects of ethanol [23]. The available experimental data accord well with clinical studies in which ethanol abuse has come to be interpreted as a compensation for inherent deficits in the endogenous opioid system, and opioid antagonists have been found useful in preventing relapse in alcoholics [29,30].

Multiple studies have also pointed out the role of opioid receptors and their endogenous ligands in psychostimulant addictions such as that of cocaine. Cocaine influences the endogenous opioid system through increase of (1) the β-endorphin [31], (2) striatal preprodynorphin mRNA [32,33,34], and (3) striatonigral dynorphin [35,36] levels in rats. In addition, the repeated administration of cocaine enhances the expression and function of opioid receptors observed as increases in the levels of MOR and KOR [37,38,39,40] without an influence on DOR density [41]. The involvement of the endogenous opioid system in the effects of cocaine was also confirmed by behavioral studies that clearly indicate that KORs are involved in the motivational and rewarding effects of cocaine [42,43,44,45]. In contrast, the data about MOR contribution in the rewarding effects of cocaine are conflicting.

Some studies [46,47] have reported that the motivational effects of cocaine measured in the locomotor sensitization paradigm were abolished in MOR knockout mice. In contrast, Becker et al. [48] observed no change, while Hall et al. [49] noted even enhanced cocaine-induced locomotor sensitization in this group of animals. Differences were also observed for the rewarding effects of cocaine measured in the conditioned place preference (CPP)test, and they presumably were caused by variations in dose and experimental conditions (number of pairings or number and duration of conditioning sessions). Most published experiments with MOR knockout mice have indicated the maintenance of cocaine CPP in this group [49,50]. The exception is the reduction of cocaine CPP observed by Hall et al. [49]. More conclusive results were obtained in experiments with MOR antagonists that attenuated the rewarding [51,52], but not motivational effects of cocaine [51]. The data regarding the role of DOR in cocaine effects are also conflicting. It has been shown that high doses of DOR antagonists block [53] or reduce [54,55,56] the rewarding effects of cocaine, while low doses did not modify both the effects of cocaine [57,58] or blocked entirely the development (but not expression) of locomotor sensitization and the conditioned rewarding effects of cocaine [59,60].

As with cocaine, pharmacological and genetic studies have demonstrated the critical role of the opioid system in several aspects of nicotine addiction. Most published studies have indicated that primarily MOR and KOR are involved in nicotine’s rewarding effects, but with opposite impact. Similar to other drugs of abuse, nicotine enhances the dopamine levels in the NAc by the activation of MOR in the VTA, and it has no rewarding effects in MOR [61], PENK gene [62], or β-endorphin [63] knockout mice. Instead, KOR and their endogenous ligands modulate the rewarding effects of nicotine in an opposing manner, which is observed as an enhancement of sensitivity to nicotine self-administration [64]. Nicotine may also indirectly activate the endogenous opioid system by increasing the release of some endogenous opioid peptides. Furthermore, it has been observed that acute nicotine administration increases plasma concentrations of β-endorphin in rats [65,66,67]. However, this effect may be rather related to a peripheral response to stress after nicotine administration than the activation of the endogenous opioid system [68,69]. In addition, chronic nicotine treatment increases the expression of mRNA for PDYN [70], while the influence on the level of PENK depends on the animal species and the regiment of administration [71,72]. The involvement of the endogenous opioid system in the effects of nicotine has also been demonstrated for withdrawal rodents and humans. Researchers have observed that the administration of an opioid receptor antagonist precipitates the nicotine withdrawal syndrome [73,74]—a decrease in MOR [61] and PENK knockout mice [62]—but not in the absence of PDYN [64] or β-endorphin [63].

The addictive properties of cannabinoids, one of the most popular stimulants, are directly related to the activation of CB1 receptors [75]. However, both pharmacological studies and genetic approaches demonstrate that cannabinoid and endogenous opioid systems interact to regulate both the neurochemical and behavioral responses to cannabinoids [76,77]. Although mechanisms underlying these interactions remain still unclear, receptors from these two systems show overlapping distribution in various brain structures. Moreover, they may form heterodimers that control GABA and glutamate release [78,79,80], and through these complexes, they regulate various behavioral responses, including drug addiction. The endogenous opioid system is involved in all phases of cannabinoid addiction—the development, maintenance [81,82,83,84], reinstatement [85], and abstinence syndrome [86]. However, Castañé et al. [81] suggest that a cooperative action between MOR and DOR is essential for the expression of cannabinoid dependence.

The endogenous opioid system plays a key role in several aspects of the addictive properties of drugs of abuse and could be an important element in identifying new possible therapeutic goals in the treatment of addiction. Moreover, anti-opioid substances may become a promising tool in the treatment of drug addictions. The introduction of all peptides with anti-opioid properties would be largely beyond the scope of this paper; thus, we will focus only on the four most commonly described: (melanocyte-inhibiting factor (MIF-1), cholecystokinin (CCK), nociceptin/rphanin FQ (N/OFQ) and neuropeptide FF (NPFF).

## 4. Anti-Opioids

### 4.1. MIF-1

The first endogenous peptide with anti-opioid properties—melanocyte-inhibiting factor (MIF-1) (Pro-Leu- Gly-NH2) was reported by Kastin et al. in 1979 [87]. Alternate forms of this peptide create a family called Tyr-MIF-1 instead of MIF-1, since Tyr-MIF-1 has been found to have its own high affinity to non-opiate binding sites in the brain [88,89,90,91]. This family of peptides includes MIF-1 (Pro-Leu-Gly-NH2), Tyr-MIF-1 (Tyr-Pro-Leu-Gly-NH2), Tyr-W-MIF-1 (Tyr-Pro-Trp-Gly-NH2), and Tyr-K-MIF-1 (Tyr-Pro-Lys-Gly-NH2), and it has been isolated from bovine hypothalamus and human brain cortex. Unlike most other putative anti-opioid peptides, they bind to MOR (with the exception of MIF-1 and Tyr-K-MIF-1) and to their own non-opioid receptors [92,93,94]. However, they are able to interact with specific receptors with about 50-fold higher affinity than with MOR [95]. This is one of the most important differences between the Tyr-MIF-1 family and other anti-opioid peptides such as CCK or NPFF—both of which possess no affinity to opioid receptors.

Most of the accumulated data for the anti-opioid properties of this group involve MIF-1 and Tyr-MIF-1, which antagonize the actions of endo- and exogenous opiates, with a higher effect observed for Tyr-MIF-1 than MIF-1 [87,90,96,97,98]. Moreover, they showed inhibitory actions not only against opioid antinociception, but also against hypothermia and opioid-induced ingestive behavior [87] with no effect on endogenous opioid independent analgesia [99]. In addition, Zadina et al. [100] have indicated that the chronic administration of opioids results in an enhancement of Tyr-MIF-1 activity. Such effect is observed only after prolonged administration and may be involved in the expression of opioid withdrawal. Similarly, Tyr-MIF-1 triggers an abstinence syndrome in morphine-dependent rodents, but not in opioid-naïve animals [93] (Table 3).

Subsequent research suggests that the anti-opioid properties of Tyr-MIF-1 seem to be mediated by MOR since this peptide shows low, but significant, affinity and selectivity for MOR. Particularly under conditions of reduced receptor reserve, e.g., in a morphine tolerant state, Tyr-MIF-1 may act as an MOR receptor antagonist. It should be emphasized that other agonists with relatively low efficiency to receptors are known to become antagonists when the efficiency of the stimulus–response coupling is reduced [126]. Moreover, correlating with their low affinity to MOR, their inhibitory effects against morphine-like activity are relatively weaker than those of traditional MOR antagonists, such as naloxone or CTOP [112].

Additionally, MIF’s peptides can act directly at opioid receptors and behave as antagonists at low concentration and as agonists at high concentrations. As expected, most of these peptides were reported to have agonistic properties for MOR in several experimental paradigms, including analgesia [101]. Here, Tyr-W-MIF-1 primarily induces dose-dependent short-lasting analgesia [127,128] and modulates gastric emptying and gastrointestinal motility [129,130]. The mentioned agonistic effects are relatively lower than those observed for morphine and are completely attenuated by MOR, but not DOR and KOR antagonists.

Nonetheless, while only a few studies have targeted Tyr-K-MIF-1 and its biological activity, its antinociceptive properties are unlikely to be related to interactions with opioid receptors, as might be for Tyr-W-MIF-1 and Tyr-MIF-1. Based on cited research, it can be clearly stated that physiologically, these peptides may biphasically, both negatively and positively, control the MOR system. Therefore, they may be considered not as anti-opioid but rather opioid-modulating peptides.

### 4.2. Cholecystokinin

Cholecystokinin (CCK) is a peptide hormone present in the gastrointestinal tract [131], but which also serves as a neuropeptide [132]. It is composed of varying numbers of amino acids depending on the post-translational modification of the 150-amino acid precursor, preprocholecystokinin. Thus, the CCK peptide hormone exists in several forms, each identified by the number of amino acids in the structure, e.g., CCK58, CCK33, CCK22, or CCK8. However, in the brain, sulfated CCK8 (CCK8S) [133] is the most abundant. This possesses one of the strongest endogenous anti-opioid properties [109]. It acts through binding to specific CCK receptors, CCKA (A for alimentary) and CCKB (B for brain) (also designated as CCK1 and CCK2) [134,135,136], and it possesses no affinity for opioid receptors [137].

CCK1 receptors were first characterized in peripheral organs such as the gallbladder, the pancreas, and the gastrointestinal tract, but they can also be found in many brain areas, mainly the interpeduncular nucleus, nucleus tractus solitarius, and area postrema [138,139]. It has been observed that they are principally implicated in the regulation of food and liquid intake. CCK2 receptors are localized in the gastrointestinal tract and in structures of the limbic system such as the cerebral cortex, NAc, striatum, hippocampus and amygdala, which are crucial for the control of anxiety, emotions, pain, memory, and satiety [140,141].

Initially as a result of a similarity to gastrin, interest in CCK has been focused on its role in the control of food intake. Subsequently, the discovery of the various effects of CCK on the central nervous system increased the interest in the brain and led to a number of studies associating with the central effects of this peptide. The anti-opioid activity of CCK was first reported by Faris et al. [107]. Afterward, it was confirmed and characterized in a large number of behavioral studies (Table 3). However, the cellular and molecular mechanisms underlying the CCK anti-opioid action are still poorly understood. The presence of opioid receptors on CCK-containing neurons suggests the direct effect of opioids on the release of CCK [142] with differences at the central nervous system and spinal cord level. In the central nervous system, chronic opioid administration increases CCK release and CCK2 receptor expression [136,143,144]. In contrast, at the spinal cord level, both selective MOR (DAMGO) and low doses of DOR (DTLET) agonist result in a reduction of CCK release in a naloxone-reversible manner [145]. Furthermore, the antagonism of endogenous CCK at the spinal cord level may enhance the effects of exogenously administered opioid receptor agonists [146]. The observed effect may indicate that endogenously released CCK tonically inhibits opioid activity and reduces the binding affinity of MOR ligands [137,147]. It also has been suggested that endogenous CCK modulates the potency of spinal opioids observed in inflammatory or neuropathic pain [146,148]. Moreover, in contrast to NPFF and N/OFQ, after central and peripheral administration, CCK reduces the antinociception evoked by exogenous, as well as endogenous opioids, presumably by the activation of CCK2 receptors [113]. However, unexpectedly, mice lacking the CCK2 receptor showed a lower pain threshold than wild-type animals and are less sensitive to the analgesic effects of endogenous opioids or morphine [113]. It should also be emphasized that the stimulation of CCK1 receptors facilitates pain control by the opioid system [135].

It is increasingly apparent that CCK regulates a variety of physiological processes, including cognition, reward, and learning or memory [149,150]. Based on the localization of CCK receptors within the brain reward system, experiments clearly indicate that endogenous CCK is necessary for the morphine rewarding effects [151] and is upregulated after chronic morphine treatment. This has been confirmed by subsequent studies that have demonstrated that CCK receptor blockade or knockout inhibits the development of opioid tolerance, dependence, withdrawal syndrome [134,152], and the reinstatement of morphine reward [153,154]. At the same time, it has been noticed that the administration of exogenous CCK-8 prevents the development of morphine addiction and reduces withdrawal symptoms [155]. Crespi et al. have reported that CCK is also involved in cocaine [156] and ethanol [157] intake. Some researchers explain these effects as the result of CCK-mediated regulation of anxiety and stress [158] or food and liquid consumption [159]. However, mostly, it is concluded that this effect points out a direct implication in the drug-dependence phenomenon by interaction with other neurotransmitter systems. It is indeed well documented that neurotransmitters such as dopamine and serotonin are largely involved in drug abuse and that these systems can interact with central CCK functions. However, in current thinking, the main focus of the anti-opioid action of CKK remains the CCK–opioid interaction that at least in the rodent is mediated by the CCK2 receptors [160,161,162].

### 4.3. Nociceptin/Orphanin FQ (N/OFQ)

In 1994, soon after the cloning of classical opioid receptors, several research groups identified a new G protein–coupled receptor with high homology to opioid receptors, but which was not able to bind opioid ligands. On this basis, it was named opioid receptor-like-1 (ORL-1) [163]. One year after the cloning of the NOP receptor, two groups independently identified the endogenous ORL1-ligand, named “nociceptin/orphanin FQ” (N/OFQ). The name “nociceptin” was determined on the basis of its ability to elicit hyperalgesia after supraspinal administration in mice, and “orphanin FQ” was chosen for its ability to recognize a previously known orphan receptor, and for its first and last amino acid residues (F and Q) [164,165]. Following the identification of N/OFQ as the endogenous agonist of ORL-1, the receptor was renamed “nociceptin opioid peptide receptor” and abbreviated as “NOP receptor”. International Union of Basic and Clinical Pharmacology (IUPHAR) considers it a subcategory of the opioid peptide receptor family [166].

N/OFQ displays a primary sequence very similar to that of opioid peptides, especially Dynorphin A. However, Phe being presented in position 1 instead of Tyr makes this peptide highly selective for its own receptor over classical opioid receptors [167], and its pharmacological effects are not reversed by the non-selective opioid antagonist—naloxone [114]. Since its discovery, N/OFQ has been of great interest to researchers as a result of its wide distribution in the nervous system. It is found in the cortex, anterior olfactory nucleus, lateral septum, hypothalamus, hippocampus, amygdala, central gray, pontine nuclei, interpeduncular nucleus, substantia nigra, raphe complex, locus coeruleus, and spinal cord [168].

The available results show that N/OFQ evokes complex pharmacological effects in animals, eliciting either an anti-opioid/anti-hyperalgesic action or analgesic effect depending on the dose and route of administration, testing paradigm, and even animal species. Functionally, the N/OFQ is considered to have an anti-opioid action (Table 3). When injected intracerebroventricularly (i.c.v), it antagonizes opioid analgesia, including opioid-mediated stress-induced analgesia and the analgesic effects of MOR, DOR, and KOR agonists [115]. In contrast to i.c.v. injection, spinal (i.t.) administration of this peptide produces an opioid-like, antinociceptive effect [169]. However, the synthetic NOP agonist, Ro65-6570, given systemically, did not produce statistically significant effects in the mouse tail withdrawal assay, but it was effective in inhibiting the nociceptive effect in inflammatory pain [170,171]. This antinociceptive effect was observed at a dose that induced severe motor side effects [170]. In turn, systemic administration Ro 65-6570 to non-human primates exerted potent and efficacious antinociception with the absence of motor and sedative side effects [152]. More importantly, this agonist did not produce reinforcing effects that were per se comparable with opioid agonists [172]. Additionally, mice knockout for the NOP gene displayed increased nociceptive behaviors in some assays (i.e., formalin test) [171].

Furthermore, N/OFQ administrated i.t. enhances morphine—or electroacupuncture—induced analgesia [169,173,174], and it produces antinociceptive synergy with morphine in experimental neuropathy [119] in rodents. Moreover, the systemic or i.t. coactivation of MOP and NOP receptors produced synergistic antinociception in primates [116,175] without other side effects. These studies strongly support the therapeutic potential of mixed MOP/NOP agonists as innovative analgesics.

Therefore, the consequence of this research was the introduction of cebranopadol, a first-in-class potent analgesic agent with agonistic activity that targets NOP and opioid receptors [176]. This compound displays analgesic, antiallodynic, and antihyperalgesic properties in several rat models of acute nociceptive, inflammatory, cancer, and neuropathic pain [177,178]. In contrast to classic opioids, it has a higher analgesic potency in models of neuropathic pain than in acute nociceptive pain [179]. In addition, even at higher doses, cebranopadol does not induce motor coordination deficits or respiratory depression [177] and has limited potential to produce opioid-type physical dependence in rodents [180]. Hence, it seems to possess a broader therapeutic window than classical opioids. Therefore, this compound is particularly interesting as a novel, potent bifunctional agonist of NOP/opioid receptors.

Research indicates that the interactions between N/OFQ and the opioid system are responsible for maintaining homeostasis in the body. Indeed, Yuan et al. show that the chronic administration of high doses of morphine accelerates the release and biosynthesis of N/OFQ in the rat brain. This effect is presumably involved in the blockade of the opioid analgesic effect and promotes the development of morphine tolerance. Surprisingly, the chronic administration of exogenous N/OFQ, concomitantly with morphine, attenuates the development of morphine tolerance, without impact on the basal and morphine nociceptive responses after single administration [181]. These observations may indicate that N/OFQ is not implicated in the early phase of the development of tolerance.

Increasing evidence suggests that this peptide may also play a crucial role in the regulation of reward mechanisms and drug abuse processes. As with other anti-opioid peptides, receptors for N/OFQ are widely represented in areas implicated in drug abuse and reward. The injection of N/OFQ results in the blockade of the rewarding properties of several common drugs of abuse such as morphine, cocaine, amphetamines, or ethanol [120,182,183,184,185,186,187,188] without side effects per se [189]. Interestingly, although N/OFQ has been proposed to be a functional “anti-opioid peptide”, it does not exhibit the aversive properties typical for opioid receptor antagonists. Based upon the ability of N/OFQ to block extracellular dopamine levels and block the CPP of so many abused drugs, it is surprising that N/OFQ was ineffective in attenuating heroin self-administration in rats [118] but was successful in blocking ethanol self-administration [190,191,192]. However, some studies found N/OFQ agonists decrease the self-administration of ethanol only in rats with a history of ethanol dependence, but not in naïve rats [193,194]. There is also no information demonstrating the influence of N/OFQ on the self-administration of cocaine or nicotine. It also should be noted that the effect of N/OFQ receptor activation was found to be different in two standard “drug abuse” paradigms, CPP and self-administration [195]. Presumably, N/OFQ receptor agonists are effective in attenuating acquisition but not the expression of the drug reward effect that underlies the self-administration paradigm. This suggests that different mechanisms may control these two paradigms. There is also evidence that N/OFQ can modulate the opioid withdrawal syndrome, thus decreasing the risk of relapse, and it may facilitate the treatment of opioid addiction. This statement is supported by behavioral studies that have uncovered that N/OFQ inhibits naloxone precipitated morphine withdrawal [196] without any change in behavior of the morphine-dependent animals when given alone [169]. The effect N/OFQ on the locomotor activity in rodents is still unclear, since neither a reduction nor stimulation has been observed. Moreover, the results were dependent on the dose and whether the animals were habituated or not to the test environment [197,198]. However, it has been reported that N/OFQ does not alter locomotor sensitization induced by cocaine and morphine [199]. Therefore, this outcome supports the idea that N/OFQ does not interfere with the motivational effects of drugs of abuse.

The data discussed in this review clearly indicate that N/OFQ behaves as a functional anti-opioid peptide especially in regard to the rewarding effects of opioids and ethanol. Interestingly, its influence on other physiological functions is observed after the administration of higher doses. Thus, the anti-opioid action of N/OFQ on the rewarding properties of drugs is more potent than that evoked on pain processes.

### 4.4. NPFF

Neuropeptides FF (NPFF), AF (NPAF), and SF(NPSF) are homologous, amidated peptides that were originally identified on the basis of sequence similarity to the neuropeptide FMRF-amide [184]. They have been hypothesized to have broad functions in the mammalian central nervous system, including the modulation of pain [200,201] or opiate functions [202,203], as well as the regulation of cardiovascular [204] and neuroendocrine functions [121]. One of these sequences, NPFF (FLFQPQRF-NH2), has been characterized as an endogenous anti-opioid peptide [122,124]. This peptide evokes pharmacological effects by interacting with the G-protein-coupled receptors, NPFF1 and NPFF2 [205,206], which are widely expressed in spinal cord and brain regions regulating emotional functions (fear, anxiety, reward) [207,208]. It should be emphasized that NPFF does not bind to opioid receptors, and conversely, opioids have no measurable affinity toward NPFF receptors [123,209]. However, many studies suggest that the NPFF system is functionally coupled to the opioid system, and each can mutually interact [210]. Furthermore, remarkable similarities in several key anti-opioid actions between CCK and NPFF were noted. Both peptides administrated alone cause no significant pronociceptive effects; rather, they exert a dose-dependent antagonism of opioid-induced analgesia. Furthermore, both peptides and their receptors strategically overlap with the opioid receptor distribution in many brain regions related to pain perception. Finally, the NPFF and CCK system can be upregulated and mobilized to exert an anti-opioid action in response to enhanced opioidergic transmission [112].

It has been observed that NPFFs possess a bimodal effect on pain perception: an anti-opioid effect after supraspinal administration and an opioid-like effect at the spinal level [211], similarly to N/OFQ. In general, NPFF reverses morphine- and stress-induced analgesia after i.c.v. administration [200,203,212]. In addition, pretreatment with anti-NPFF-IgG restores the analgesic effects of morphine in morphine-tolerant rats [213] and potentiates opioid analgesia [214]. These findings, along with the observations that long-term NPFF administration decreases the density of the MOR [124], suggests that these receptors are under the tonic inhibitory control of NPFF. NPFF also seems to play a role in opiate tolerance, since it has been observed that anti-NPFF antibodies can reverse the development of tolerance to morphine [215], as well as reduce the symptoms of naloxone-induced withdrawal syndrome [213,216]. Moreover, the chronic administration of morphine increases concentrations of NPFF in the cerebrospinal fluid [217,218] and presumably may underlie the development of opioid tolerance that is associated with increased NPFF activity. Furthermore, its action does not directly depend on opioid receptors. This effect also appears to be responsible for the occurrence of opioid withdrawal syndrome [202]. On the other hand, NPFF and its analogs, which are administered i.t., induce long-lasting analgesia, probably by increasing opioid peptides release in the spinal cord through the blockade of DOR autoreceptors [219], and they also potentiate morphine-induced analgesia [201,211].

Nociception is the physiological function in which this interaction between NPFF and opioids has been the most extensively studied; notwithstanding that reward, locomotion, feeding and intestinal motility are also affected. The presence of both NPFF-like immunoreactive material and NPFF binding sites in the mesolimbic structures [209,220] may point toward the involvement of NPFF in drug addiction processes. In fact, it also suggests that NPFF may be a good candidate for directly or indirectly regulating mesocorticolimbic system activity. This is because the supraspinal administration of NPFF or analogs antagonizes the rewarding effect of morphine by inhibiting dopamine release in the mesolimbic reward system [221,222]. In addition, Kotlinska et al. found that NPFF is able to block the expression of the rewarding effects of cocaine and amphetamine in the CPP test [223].

In contrast to the above results, NPFF is not able to inhibit the expression of ethanol-induced CPP. However, it should be noted that NPFF neither induced place preference nor aversion, although a tendency to aversive effect was seen after the administration of NPFF at a high dose. Several studies also showed the influence of NPFF on the motivational effects of drugs of abuse. This is observed as an inhibition of the hiperlocomotor effect. In fact, NPFF, administrated i.c.v. reduces the locomotor activity triggered by exposure to novelty and decreases dose-dependently the potentiation of novelty-induced locomotor response degradation [207]. Furthermore, NPFF i.c.v. administration reduces the sensitization to hyperlocomotor effects that is crucial in the development of morphine [220,222], heroin [224], cocaine [225], as well as amphetamine [223] and ethanol dependence [222]. These data show that NPFF receptors may be involved not only in the rewarding, but also in the motivational effects of many drugs of abuse. However, it should be emphasized that NPFF is unable to inhibit the rewarding effects of ethanol. It is probable that this effect is due to the fact that ethanol reward is a more complex process, and apart from the role of opioids, many other neurotransmitters are also involved in this effect.

In addition, one of the newly discovered kisspeptin derivatives, kissorphin (KSO) (Tyr-Asn-Trp-Asn-Ser-Phe-NH2), also evokes an anti-opioid effect. This peptide does not possess any activity on the GPR-54 receptor and does not affect the release of GnRH [226]. However, it activates the receptors for the anti-opioid peptide, NPFF. The NPFF-like anti-opioid activity of KSO was supported by Simonin et al. [226] and Milton [227], who showed that the biological activity of the KSO peptide is antagonized by the NPFF receptor antagonist RF9 but not the GRP-54 receptor antagonist KP234 [227]. Recent data [228,229,230] also confirmed the NPFF-like activity of KSO and provide a support for the anti-opioid character of KSO which, in contrast to NPFF, inhibits rewarding and motivational effects of ethanol.

## 5. Anti-Opioid Peptides: Potential Therapeutic Interest

The treatment of drug addiction still remains a medical need due to the dearth of effective and approved pharmacotherapies. The assessment of current addiction treatment has revealed the stark reality of the limited options to treat opioid and psychostimulant abuse. A growing problem is the elimination of withdrawal symptoms, as these often lead to a return to addiction even after a long period of abstinence. It is considered that the discovery of a group of substances with anti-opioid activity creates advanced therapeutic possibilities, and their use may result in the introduction of completely new and safer methods of addiction treatment.

In fact, the ability of N/OFQ to block the rewarding effects of opioids and some other psychostimulants, as well as its anxiolytic profile, underlines its therapeutically beneficial profile for drug abuse and relapse treatment. This is particularly important from a therapeutic point of view, since opioid antagonists such as naltrexone, which is currently used for the treatment of ethanol and opioid addiction, possess many drawbacks. Among these are anxiogenic activity and the precipitation of withdrawal symptoms. In contrast, N/OFQ does not exhibit the aversive properties at doses that block the rewarding properties of morphine and ethanol. Moreover, in contrast to opioid antagonists, the activation of brain NOP receptors attenuates alcohol [231] and opioid [232] withdrawal symptoms in rats.

Preclinical studies in rodents and non-human primates have shown that non-peptide, small-molecule N/OFQ receptor agonists demonstrate efficacy in attenuating the rewarding effects of various abused drugs. These compounds (AT-312, Ro 64-6198) show promising impact on the rewarding effects of many drugs of abuse and may have potential as pharmacotherapy even for treating polydrug addiction [192,233]. However, the stimulation of NOP receptors in the mesolimbic circuitry may lead, for example, to a hypodopaminergic and hypohedonic state that can increase the motivation for drugs of abuse. In fact, published data show that the activation of NOP following intra-ventral tegmental area (VTA) administration of N/OFQ attenuates dopamine release in the nucleus accumbens [184]. On the other hand, the administration of NOP agonists may depress N/OFQ transmission through receptor desensitization [234], and, in such a way, it may result in paradoxical antagonistic effects.

Thus, another strategy to attenuate drug abuse is to focus on NOP blockade. For instance, BTRX-246040 (also known as LY2940094), a selective and potent NOP antagonist [235], was found to reduce alcohol consumption in two different lines of genetically selected alcohol preferring rats [236] and in clinical trials with patients diagnosed with alcohol use disorders [237]. The putative therapeutic potential of NOP antagonists comes from studies in genetically modified NOP knockout rats. These animals self-administered significantly smaller amounts of alcohol, cocaine, and heroin, but showed unimpaired motivation for saccharin, which is a natural reward [238]. Hence, this group of compounds is more effective in preventing drug abuse [239] than NOP agonists and has promising utility for treating drug addiction.

Finally, a potentially effective alternative strategy for treatment of drug abuse is the coactivation of NOP and MOP receptors. Ciccocioppo et al. [240] found that buprenorphine, a partial agonist at MOP [240] and NOP receptors [241,242], has the ability to attenuate ethanol consumption in rats at high concentrations. Moreover, unlike buprenorphine, certain recently developed bifunctional NOP/MOP partial agonists (i.e., BU08028, BU10038, and AT-121) do not exhibit reinforcing effects in non-human primates [243,244,245]. These compounds selectively attenuate alcohol intake (BU08028) [246] and significantly attenuate oxycodone-induced reinforcing effects (daily systemic AT-121 pretreatment) [244] without disrupting food-maintained operant behavior. Furthermore, recently, two independent studies have demonstrated that cebranopadol, another NOP and MOP receptor agonist, is efficacious in attenuating the motivation for cocaine in drug self-administration studies, while leaving unaffected (or slightly increased) the consumption of natural rewards [247,248]. Therefore, it is possible that mixed NOP/MOP partial agonists may have great therapeutic potential for treating substance use disorders and/or polydrug addiction [249] with fewer side effects.

The broad spectrum of actions of N/OFQ has provoked the interest of academic and industrial researchers and generated a large panel of N/OFQ receptor ligands useful for target validation studies. In fact, N/OFQ receptor agonists have been proposed as innovative drugs for treating hypertension [250] and urinary incontinence [251], whereas N/OFQ receptor selective antagonists have been suggested as novel treatment for Parkinson’s disease [252], depression (including BTRX-246040, an NOP antagonist already tested in humans) [253,254], and possible drug abuse [239]. BTRX-246040 was also found to reduce depression symptoms in a second trial with heavy alcohol drinkers. Given the comorbidity of major depression and alcohol use disorder, a compound with such effects in patients could be a valuable addition to the medications available [255]. In addition, NOP antagonists have already been used to identify novel conditions/diseases for which the blockade of NOP receptor is associated with beneficial effects (traumatic injuries of the central nervous system, post-traumatic stress disorders, sepsis) [255,256,257,258,259].

In turn, some behavioral experiments have revealed that opioid-N/OFQ hybrids such as SR16435, which behaves as a mixed MOP–NOP receptor partial agonist [260], are highly promising analgesics for the treatment of acute and even neuropathic pain, devoid of respiratory depression [261]. For example, a mixed opioid/NOP agonist, cebranopadol, is in early phase clinical development in treating diabetic neuropathy, cancer pain, and lower back pain [262,263,264].

N/OFQ appears to have the widest potential clinical use, which is also directly related to its pleiotropic/multidirectional activities; however, likewise, CCK2 receptor antagonists have displayed a preventive or therapeutic effect on morphine dependence and withdrawal [265]. Moreover, observation that the CCK2 receptor plays a crucial role in the induction and persistence of anxiety and major depression has led to using CCK-4 routinely to induce anxiety in behavioral models. Additionally, CCK compounds, especially the selective CCKB antagonists, may be interesting drugs in the management of pain. Indeed, even if they do not induce analgesic responses alone, they are able to potentiate the antinociceptive effects of the opioids and reduce the eventual side effects of opioid chronic treatment. Thus, a concomitant administration of morphine and a CCK antagonist may permit reduction of the dose and frequency of morphine administration required for pain relief [266,267]. CCKB antagonists may also be useful in reducing the morphine withdrawal effect [268].

Regarding MIF-1, its high stability in human blood, combined with its long-lasting biological activity, makes this peptide a valuable candidate for a human therapeutic agent [269,270]. However, presently, this peptide is rather considered as a prototype for the group of anti-opioid peptides.

Other promising therapeutic candidates for pain and abuse treatment are NPFF and KSO. The last is a novel NPFF receptor agonist. In fact, previous studies using these peptides have provided a better understanding of the processes involved in ethanol, morphine, cocaine, and amphetamine dependence. However, they are still used mainly as reference substances in behavioral experiments and are the basis for the search for new drugs.

The use of anti-opioid peptides in behavioral studies has significantly contributed to a better understanding of the processes that lead to the development, maintenance, and relapse of drug addiction. However, for most of them, a restriction to widespread use is the difficulty of administration, since most of these compounds are unable to penetrate the blood–brain barrier after peripheral administration and thus cannot induce central effects. In order to overcome this limitation, further research is needed to develop new compounds of a non-peptide nature with anti-opioid activity.

## 6. Conclusions

The pharmacological effects of acute and chronic opioid administration could be modulated or even blocked by several endogenous peptides. All these peptides are referred to as anti-opioid or to a lesser extent, as opioid-modulating peptides, since some of them also possess pharmacological opioid-like properties. However, it should be emphasized that no single peptide possesses naloxone-like effects to all systems. Indeed, a compound that would be capable of modulating the multiple behavioral effects of the endogenous opioids should itself contain many elements. The variety of endogenous opiates strengthens our idea that endogenous anti-opioids constitute a numerous and diverse group, and the MIF-1 related peptides CCK, N/OFQ, and NPFF represent distinct neuronal systems.

The confirmation that endogenous opioid-modulating peptides may control the pharmacological effects of morphine and other drugs of abuse opens new, broad prospects for their therapeutic use, notably for alleviating pain and for treating opioid abuse. These compounds possess high efficacy, and some of them are already at the clinical trial stage. However, the precise mechanisms involved in these effects are still not completely characterized, and anti-opioids still remain a relatively underexplored system with potential therapeutic importance.

## Figures and Tables

**Table 1:** Opioid receptors and their functions.

Opioid Receptor	Subtypes	Previous and Unofficial Names	Effects of Activation
µ	µ_1_, µ_2_, µ_3_	Mu receptor/MOP/OP3/MOPr/opioid receptor, mu 1	spinal and supraspinal analgesia respiratory and cardiac depression euphoria sedation physical dependence tolerance changes of smooth muscle tone decreased gastrointestinal motility urinary retention pruritus
δ	δ_1_, δ_2_	DOP/DOR/OP1/Delta receptor/DOR-1/DOPr	spinal and supraspinal analgesia without respiratory compromise antidedepressant effect antianxiety effect decrease of colonic transit time
κ	κ_1_, κ_2_, κ_3_	KOR-1/Kappa receptor/OP2/KOP/KOPr	spinal and supraspinal analgesia miosis psychotomimetic effects (dysphoria, agitation) sedation without pronounced respiratory depression, euphoria or gastrointestinal effects
Nociceptin receptor	ORL1	N/OFQ receptor/OP4/KOR-3/NOCIR/kappa3-related opioid receptor/MOR-C/nociceptin receptor ORL1/XOR1/NOP-r/nociceptin/orphanin FQ receptor/NOPr	analgesia at the spinal level reduction of locomotor activity impairment of memory increase of food intake anxiolytic effect increase of water diuresis stimulation of immune response

**Table 2:** Endogenous opioid peptides, their precursors, and receptor affinity.

Precursor	Endogenous Opioid Peptide	Relative Opioid Receptor Affinity
Proenkephalin (PENK)	[Met]-enkephalin [Leu]-enkephalin	µ, δ (δ >> µ)
Proopiomelanocortin (POMC)	β-endorphin	µ and δ (δ = µ)
Prodynorphin (PDYN)	Dynorphin A Dynorphin A (1–8) Dynorphin B α-neoendorphin β-neoendorphin	κ, µ, δ (κ >> µ, δ)
Prepronociceptin (PNOC)	Nociceptin/Orphanin FQ	ORL-1
Unknown	Endomorphin-1 Endomorphin-2	µ

**Table 3:** Attenuation of opioid effects by selected endogenous neuropeptides.

Peptide	Effect	References
**MIF-1** 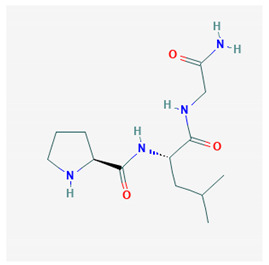 PubChem Identifier: CID 92910 URL: https://pubchem.ncbi.nlm.nih.gov/compound/92910	Attenuates morphine antinociception	[87,96,97,101,102,103,104]
	Attenuates stress-induced antinociception	[87,99,104,105,106]
	Attenuates enkephalinergic analgesia	[105]
	Attenuates morphine-induced hypothermia and inhibit guinea pig ileum contractions	[106]
	Precipitate morphine withdrawal symptoms	[93]
	Inhibition of hypothermia and hypomotility produced by morphine	[106]
**CCK-8** 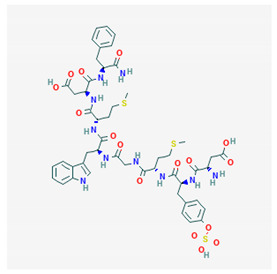 PubChem Identifier: CID 9833444 URL: https://pubchem.ncbi.nlm.nih.gov/compound/9833444	Attenuates morphine antinociception	[107,108,109,110]
	Attenuates foot shock antinociception	[107,110]
	CCK-8 antagonist attenuates morphine tolerance	[111,112]
	CCK-8 antagonist potentiates analgesia morphine	[111]
	CCK-8 antagonist does not block morphine dependence	[113]
	Attenuates β-endorphin (1-31) catalepsy	[113]
**Nociceptin/Orphanin FQ** 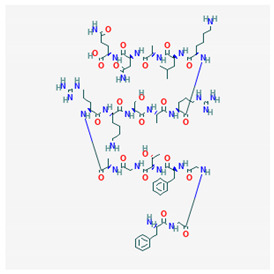 PubChem Identifier: CID 16131448 URL: https://pubchem.ncbi.nlm.nih.gov/compound/16131448	Attenuates morphine antinociception	[114]
	Inhibits morphine-induced CPP	[115,116,117,118]
	Inhibits ethanol-induced CPP	[105,115]
	Blockade or deprivation of NOP receptors potentiate rewarding effects of morphine	[119]
**NPFF** 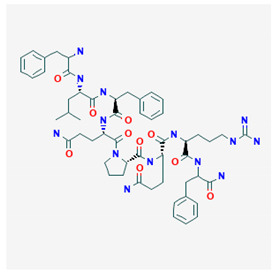 PubChem Identifier: CID 123797 URL: https://pubchem.ncbi.nlm.nih.gov/compound/123797	Precipitates opioid withdrawal syndrome	[118]
	Attenuates morphine antinociception	[120]
	Chronic morphine increases NPFF levels in cerebrospinal fluid	[118,121,122]
	Anti-NPFF IgG attenuates naloxone-induced	[118]
	Anti-NPFF IgG reverses morphine tolerance in the rat	[123]
	Putative NPFF antagonist attenuates morphine	[124]
	Suppresses DAMGO-induced inhibition of withdrawal abstinence syndrome	[125]
